# Monogenic Versus Multifactorial Inheritance in the Development of Isolated Cleft Palate: A Whole Genome Sequencing Study

**DOI:** 10.3389/fgene.2022.828534

**Published:** 2022-02-24

**Authors:** Baiba Lace, Sander Pajusalu, Diana Livcane, Ieva Grinfelde, Ilze Akota, Ieva Mauliņa, Biruta Barkāne, Janis Stavusis, Inna Inashkina

**Affiliations:** ^1^ Latvian Biomedical Research and Study Centre, Riga, Latvia; ^2^ Department of Clinical Genetics, Institute of Clinical Medicine, Faculty of Medicine, University of Tartu, Tartu, Estonia; ^3^ Department of Clinical Genetics, United Laboratories, Tartu University Hospital, Tartu, Estonia; ^4^ Cleft, Lip and Palate Center, Institute of Stomatology, Riga Stradins’University, Riga, Latvia; ^5^ Medical Genetics Clinic, Children’s Clinical University Hospital, Riga, Latvia

**Keywords:** isolated cleft palate, whole genome sequencing, rare monogenic diseases, recurrence risk, PCGF2

## Abstract

Craniofacial morphogenesis is highly complex, as is the anatomical region involved. Errors during this process, resulting in orofacial clefts, occur in more than 400 genetic syndromes. Some cases of cleft lip and/or palate (CLP) are caused by mutations in single genes; however, complex interactions between genetic and environmental factors are considered to be responsible for the majority of non-syndromic CLP development. The aim of the current study was to identify genetic risk factors in patients with isolated cleft palate (CP) by whole genome sequencing. Patients with isolated CP (*n* = 30) recruited from the Riga Cleft Lip and Palate Centre, Institute of Stomatology, Riga, were analyzed by whole genome sequencing. Pathogenic or likely pathogenic variants were discovered in genes associated with CP (*TBX22*, *COL2A1*, *FBN1*, *PCGF2*, and *KMT2D*) in five patients; hence, rare disease variants were identified in 17% of patients with non-syndromic isolated CP. Our results were relevant to routine genetic counselling practice and genetic testing recommendations. Based on our data, we propose that all newborns with orofacial clefts should be offered genetic testing, at least for a panel of known CLP genes. Only if the results are negative and there is no suggestive family history or additional clinical symptoms (which would support additional exome or genome-wide investigation), should multifactorial empiric recurrence risk prediction tools be applied for families.

## Introduction

Craniofacial morphogenesis is a highly complex process, reflecting the sophistication of the anatomical region it concerns. Impairment of this process, resulting in orofacial clefts, occurs in more than 400 genetic syndromes, with various modes of inheritance (OMIM, 2020). Some cases of cleft lip and/or palate (CLP) are caused by mutations in single genes, including *COL11A1*, *GRHL3*, *IRF6*, *MSX1*, *TBX22*, or *TP63*, with 30–40 genes typically screened in CLP clinical diagnosis gene panels (ConcertGenetics, 2021).

In 2008, it was reported that mutation screening of >20 candidate CLP genes resulted in identification of potential syndromic CLP-causing mutations in <10% of screened individuals (Vieira, 2008). Further, complex interactions between genetic and environmental factors mediate non-syndromic CLP development and are responsible for the majority of non-syndromic CLP cases (Fogh-Andersen, 1971).

Mechanisms leading to the development of isolated cleft palate (CP) are thought to differ from those leading to CLP, as a median defect; this classification was proposed in 1971 and remains valid to date (Fogh-Andersen, 1971). Isolated cleft palate is a congenital anomaly caused by the failure of secondary palate fusion process, which is anatomically distinct from the primary palate development. Formation of secondary palate starts around seventh gestational week and continues up to the 12th gestational week, meanwhile, primary palate formation is already finished by seventh/8 weeks (Mossey et al., 2009). CP is less frequent developmental defects compared to CLP. CP prevalence is 1.3–25.3 per 10,000 births and it differs between countries. Higher prevalence registered in Canada and some parts of Northern Europe, lower prevalence in South America and Africa. CLP prevalence is 3.4–22.9 per 10,000 births and it is higher in South America and Asia, thus emphasizing the difference in population genetics. CP is more common in females contrary to CLP, which is more frequent in males, and this aspect is constant across all populations (Mossey and Modell 2012). The manifestation of CP severity varies, it can be classified as unilateral or bilateral, complete, or incomplete, and a submucous form of cleft palate. European congenital anomaly registry EUROCAT study revealed that in 18% cases cleft palate was associated with other congenital anomalies, and in 27% cases CP is one of the symptoms in recognized genetic syndromes. CP and CLP accompanying other major congenital anomalies are referred as syndromic forms, and, similarly, CP and CLP not connected with major congenital anomalies are non-syndromic types (Calzolari et al., 2007). Human Phenotype Ontology database currently lists 857 disease associations with a cleft palate, which includes monogenic diseases with known or unknown etiology, chromosomal aberrations, imprinting disorders and embryofetopathies. CP caused by teratogenic exposure includes use of certain medications during pregnancy, like aminopterin, hydantoin and isotretinoin, or maternal abuse of alcohol, as well as pregnancies complicated by gestational diabetes (Köhler et al., 2021). By far the most common genetic syndrome associated with orofacial cleft is van der Woude syndrome type 1 and 2, caused by pathogenic variants in *IRF6* and *GRHL3* genes, accordingly. Van der Woude syndrome, however, is a rare exception, as both forms CLP and CP can be present in this syndrome, along with the lip pits (Online Mendelian Inheritance in Man, 1960).

Genetic studies focused on case (or patient parent trio)-control association studies, and facilitated identification of CLP candidate genes, supporting the theory of predominantly multifactorial inheritance patterns in such patients. Complex disease research requires high statistical power, therefore frequently genetic studies included CLP and CP cases as a one sample group. Selected examples of genes whose minor effect variants contribute to CLP development include *TGFA* (Letra et al., 2012)*, IRF6* (Murray et al., 1990), *TGFB2*, *MSX1*, *and TGFB3* (Lidral and Shiang 1997)*, TBX22* (Marçano et al., 2004), *GRHL3* (Peyrard-Janvid et al., 2014) *CTNND1*, *PLEKHA7*, *PLEKHA5* (L. L. Cox et al., 2018) and *GDF11* and *FST* (T. C. Cox et al., 2019)*.* Further, some association studies identified only risk loci lacking known genes, namely chromosomes 9q21 and 2q32 (Marazita et al., 2004), 19q13 (Warrington et al., 2006) and 8p11 (Riley et al., 2007). Ludwig et al. conducted a large meta-analysis of informative cohorts, leading to detection of seven new loci, in addition to those already known, as follows: chromosomes 1p36, 2p21, 3p11.1, 8q21.3, 13q31.1, 8q24, and 15q22 (Ludwig et al., 2012).

More affordable genetic technologies have transformed CLP research; most association studies are now genome-wide, and three large studies employing whole exome and whole genome approaches have been conducted. Whole exome sequencing (WES) of 57 multinational families was conducted with the precise aim of identifying novel copy number variations in known genes and risk loci, and detected five informative deletions in the *CDH1*, *FGF8*, *FGFR4*, *TRPS1*, and *FTCD* genes (Bureau et al., 2014). To date, the largest study conducted involved whole genome sequencing (WGS) of 315 European and 265 Latin American trios, all having offspring with CLP, resulting in genome-wide association data confirming previous findings and adding a new locus, chromosome 21q22.3 (Mukhopadhyay et al., 2020).

Despite massive WGS studies, it has always been presumed that some patients with non-syndromic CLP have rare variants in disease-causing genes. This hypothesis was confirmed by Basha et al., who conducted a WES study demonstrating that approximately 10% of cases previously described as non-syndromic CLP were patients with rare genetic syndromes. Further, patients were first screened for *IRF6* mutations, and positive cases were excluded from the study; therefore, the actual percentage with genetic syndromes may have been higher (Basha et al., 2018).

Our aim in the current project was to identify genetic risk factors by WGS in patients with isolated CP. We hypothesized that we would identify rare variants causing monogenic syndromes, as well as new copy number variations, in a well-described group of patients with isolated CP.

## Materials and Methods

### Recruitment of Families and Ethics Statement

Patients were recruited in 2000–2020 from the Riga Cleft Lip and Palate Centre, Institute of Stomatology, Riga Stradins University as described previously (Lace et al., 2006). All patients with orofacial clefts were evaluated by clinical geneticist and initial investigations of syndromic forms were performed. 56 probands with isolated cleft palate and both of their parents were included in the study, of them 21 cases were rejected due to insufficient DNA quality because of long term storage and multiple episodes of thaw and re-freezing. Five patients with known pathogenic variants in *IRF6* and *GRHL3* genes were excluded from the study as syndromic forms of cleft palate cases. In the families with identified *de novo* variants DNA profiling was performed.

This study was approved by the Central Medical ethics committee of Latvia. The study was performed in accordance with the ethical standards as laid down in the 1964 Declaration of Helsinki and its later amendments. All participants and/or their legal guardians provided written informed consent prior to enrolment.

### Library Preparation and Whole Genome Sequencing

Libraries were prepared from 300 ng of high-quality genomic DNA using a MGIEasy Universal DNA Library Prep Set (MGI Tech Co., Shenzhen, China), according to the manufacturer’s instructions. Libraries were sequenced at a mean coverage of 30× on the MGI MGISEQ-2000RS platform, using a nanoball approach, to generate 150 bp paired-end reads.

### Bioinformatics Analyses of Genome Data and Variant Filtering

Bioinformatics data processing was carried out at the High Performance Computing Center of the University of Tartu (University of Tartu 2018). Output Fastq files were processed and variants were called using WDL scripts and the Cromwell workflow management system (Van der Auwera and O’Connor, 2020), adhering to GATK v4 best practice guidelines. In brief, raw sequencing reads were mapped to the hg38 human reference genome using the BWA MEM algorithm (v0.7.17) (Li and Durbin 2010), followed by processing using GATK (v4.1.4.0), including flagging of duplicate reads and recalibration of base quality scores. Variants were first called for individual samples using the GATK HaplotypeCaller before multi-sample joint aggregation and re-annotation using GATK GenotypeGVCFs. The variant call set was annotated using hail .2 (Hail Team, Hail .2: https://github.com/hail-is/hail) and then uploaded to the Seqr platform (https://github.com/broadinstitute/seqr) for collaborative variant analysis. Variant filtration focused on rare variants in coding regions and splice-sites predicted to affect protein function and following the known inheritance pattern for the 198 CP associated genes (Supplementary Table S1), followed by pathogenic/likely pathogenic variants in OMIM genes, and 67 actionable genes, according to American College of Medical Genetics (ACMG) recommendations. Reported variants were validated by direct sequencing using 10 pm of each primer (primer sequences are available upon request), BigDye™ Terminator v3.1 Cycle Sequencing Kit (ThermoFisher Scientific, USA), and an ABI PRISM 3100 sequencer (Applied Biosystems, USA).

Copy number variants (CNVs) were assessed using WGS reads by two approaches. First, large CNVs and aneuploidies were screened using indexcov (Pedersen et al., 2017). Second, structural variants, including CNVs, inversions, and translocations, were called using Manta (Chen et al., 2016).

## Results

A total of 30 patients with isolated CP were analyzed by WGS, and pathogenic or likely pathogenic variants were discovered in genes associated with CP (*TBX22*, *COL2A1*, *FBN1*, *PCGF2*, *KMT2D*) in five of these patients, as detailed below. Summary of identified variants in Table 1. Here and further LAHSHAL classification was used (Kriens, 1989), where letters itself specifies anatomical site (L -lip, A—alveolus, H—hard palate, S—soft palate), capital letters identify total cleft in the anatomical site, small letter means partial cleft and star means submucous cleft.

**TABLE 1:** Identified Pathogenic/likely pathogenic variants in OMIM genes.

Gene	Variant	gnomAD allele frequency	ACMG criteria
*TBX22*	c.821_824del p.(Arg274IlefsTer21)	0	Pathogenic, PVS1, PM2, PP3
*FBN1*	c.7754T>C p.(Ile2585Thr)	1/62729	Pathogenic, PP5, PM1, PM2, PP2, PP3; ClinVar - Pathogenic/likely pathogenic
*KMT2D*	c.11905C>T p.(Gln3969Ter)	0	Pathogenic, PVS1, PM2, PP3
*COL2A1*	c.2301+1G>A p.(?)	0	Pathogenic, PVS1, PM2, PP3
*PCGF2*	c.930dup p.(Thr311HisfsTer7)	0	Likely pathogenic, PVS1, PM2

A male, age 21 years (identification no (ID): 0901) presented with a cleft soft-hard palate (HSH; LAHSHAL classification), velopharyngeal incompetence, speech disorder, and conductive deafness. An X-linked variant, NM_001109879.2 *TBX22* c.821_824del p.(Arg274IlefsTer21), was identified in the proband in a hemizygous state; his unaffected mother was a heterozygous carrier. This variant is absent from the gnomAD v3 genomes database (Karczewski et al., 2020) and classified as likely pathogenic according to the ACMG guidelines.

A 17-year-old female (ID: 13401) with maxillary hypoplasia and cleft in the soft palate was found to be a heterozygous carrier of the disease-associated variant, NM_000138.5 *FBN1* c.7754T>C p.(Ile2585Thr), inherited from her mother. This variant was previously reported in patients with Marfan syndrome (Loeys, 2001; Arnaud et al., 2019), and is listed in ClinVar as pathogenic/likely pathogenic (Landrum et al., 2018).

A female of 13-years-old (ID: 24701) with isolated CP (hSh, LAHSHAL) presented with global developmental delay, language delay, astigmatism, strabismus, and recent tension headaches. She had a history of chronic otitis media, treated by tympanoplasty. Brain MRI did not reveal any abnormalities besides vertebral artery development variation. A *de novo* disease-causing variant, NM_003482.4 *KMT2D* c.11905C>T p.(Gln3969Ter), was identified and classified as pathogenic by ACMG criteria. The variant was absent from the gnomAD v3 database (Karczewski et al., 2020). Parentity was confirmed.

A male, age 28 years, (ID: 11801) with isolated CP (hSh, LAHSHAL) was found to have a paternally inherited variant, NM_001844.5 *COL2A1* c.2301+1G>A p.(?), absent from the gnomAD v3. database and previously reported as a disease-causing alteration (Korkko et al., 2000).

A 12-year-old female patient (ID: 24801) with CP (*S*, LAHSHAL) and a history of intrauterine growth retardation, followed by developmental delay, expressive language delay, and behavioral issues, was found to have the variant NM_001136498.2 *PCGF2* c.930dup p.(Thr311HisfsTer7), also present in her affected father. This variant is absent from the gnomAD v3 database (Karczewski et al., 2020), and likely pathogenic, according to ACMG criteria. The patient had been under genetic investigation from the age of 4 years.

With the consent of patients/parents, information about identified variants was transferred to the clinical geneticist in charge. There were no other variants actionable by ACMG criteria to report back to families. Pathogenic copy number variations were not identified. In addition, 20 nucleotide polymorphisms (SNP) (Supplementary Table S2) were screened in the remaining 25 samples. SNPs included in the analysis were chosen based on a WGS study published in 2020 (Mukhopadhyay et al., 2020), and five of them were identified in our cohort; however, their allelic frequencies were not significantly different relative to gnomAD data (Karczewski et al., 2020), likely due to limited statistical power because of our small sample size (Table 2).

**TABLE 2:** Allele frequencies of the selected SNPs in study group.

Chrom locus	SNP ID or position	Gene	Variant	Allele frequency CP (current study)	Allele frequency (gnomAD-European (non-Finnish)
1p36.13	rs78998514	NA	g.18608118C>A	.430	.364
2q14.1	chr2:113,497,779	NA	g.113497779GTA>G	.136	.056
6q25.3	chr6:157,311,140	*TMEM242*	c.327 + 7608_327 + 7641del	.136	.170
8q24.3	rs72728755	*CCDC26*	n.653 + 124154A>T	.136	.1931
21q22.3	rs2839575	*PDE9A*	c.262 + 6995G>A	.613	.2749

## Discussion

Clinically available genetic tests for infants born with CLP could offer rapid and relatively inexpensive precision diagnosis, prognosis prediction, and family counselling. Arguments that some of those syndromes can be diagnosed clinically are not valid, since some of the main clinical presentations of syndromes may only emerge at a later age (e.g., Stickler syndrome); therefore, routine genetic testing is a powerful tool available for practicing clinical specialists.

One of the families included in this study had an X-linked disease-associated variant in the *TBX22* gene, which has never been described before and was inherited from the unaffected mother. *TBX22* has been described in several previous studies (Braybrook et al., 2001; Marçano et al., 2004; Demeer et al., 2018) as a cause of X-linked semi-dominant CP and ankyloglossia (CPX) (OMIM). Further, variants in *TBX22* were identified in 2.5% of isolated non-syndromic CP cases (*n* = 200) (Marçano et al., 2004), and in 16% of cases in a smaller WES study of 12 non-syndromic patients with CLP and CP (Demeer et al., 2018). Ankyloglossia is the most frequent disease presentation associated with *TBX22* gene variants and is a minor anomaly, rarely considered important to report in patients or their family members (Braybrook et al., 2001). The role of TBX22 during palatogenesis was confirmed in experiments using *Tbx22* ^*null*^ mice, which presented with submucous CP, ankyloglossia, severely underdeveloped vomer, and choanal atresia, caused by reduced bone formation in the posterior hard palate due to impaired ossification (Pauws et al., 2009). The recurrence risk calculation for our family changed because of the results of genetic testing. The mother is a carrier of this X-linked variant; therefore, the recurrence risk in the family is as high as 25%–50%, rather than the 3% empiric risk established for isolated CP. Risk of CP development in males hemizygous for the pathogenic variant is 96%, whereas in heterozygous females, the risk is only 6% (Marçano et al., 2004).

Kabuki syndrome with a *de novo* pathogenic nonsense *KMT2D* c.11905C>T, p.Gln3969Ter variant was identified in one of the probands who had global developmental delay. Cleft lip and or palate is among the structural findings in 30% of such cases (Paik and Lim 2016). The recurrence risk for the family is 1%, based on theoretical gonadal mosaicism.

A somewhat surprising finding was the identification of a patient with Marfan syndrome in our cohort. According to the Ghent criteria (Loeys et al., 2010) (Loeys et al., 2010), malar hypoplasia and a high arched palate, as present in our patient, are minor criteria for skeletal involvement. For children and young adults <20 years old, the clinical presentation of Marfan syndrome can be vague, in the absence of early cardiovascular or ocular symptoms, which explains the lack of earlier investigation for Marfan syndrome in our patient. The recurrence risk in the family is 50%.

A very rare novel disease-associated variant, c.930dup p.(Thr311HisfsTer7), in *PCGF2* was discovered in a female patient with positive family history. To date, 13 cases with pathogenic variants in the *PCGF2* gene have been described (Turnpenny et al., 2018), with prevalent features including intellectual disability, impaired growth (including intrauterine), and a range of cardiovascular and skeletal anomalies. High palate and bifid uvula have been described in two of the 13 reported patients. Our patient had a history of pre- and postnatal growth retardation, mild intellectual disability, expressive language delay, and a cleft soft palate. The *PCGF2* gene has roles in cell proliferation and differentiation and, due its dominant negative effect on histone binding during embryogenesis, it is associated with a recognizable syndrome (Turnpenny et al., 2018).

In 2010, we conducted a case-control association study of the same patient cohort with isolated CP and concluded that *COL2A1* and *COL11A2* contribute to the risk of non-syndromic CP (Nikopensius et al., 2010). Further, we hypothesized that confirmed association could also be explained by *COL11A2* and *COL2A1* disease-associated variants in some patients. The current WGS analysis confirmed the presence of a pathogenic *COL2A1* variant in a patient with CP in our study group, supporting our hypothesis that patients with monogenic disease were likely present among previously analyzed cohorts of patients with CLP. Therefore, the *COL2A1* and *COL11A2* genes are unlikely to be associated with non-syndromic forms of CP *via* complex interactions among common minor effect gene variants and environmental factors. The pathogenic variant in our case was inherited from the affected father; hence the recurrence risk for the family is 50%.

Additionally, we analyzed 20 variants identified by previous WGS association studies of individuals from Europe with CLP. Variants in the chromosomal loci 2q14.1 and 21q22.3 were more frequent in our CP patient group than in data from the general European population. Interestingly, one of these variants (c.262 + 6995G>A) is localized in the *PDE9A* gene, and haploinsufficiency of this gene and others in the 21q22.3 locus, within deletions of 1–10 Mb, is reportedly associated with a CP phenotype in 12.5% cases; however, duplications of this region of any size were never associated with cleft formation (Firth et al., 2009).

We identified rare disease variants in 17% of patients with non-syndromic isolated CP. Applications of these results in routine practice altered genetic counselling and recommendations for genetic testing. Based on our results, we propose that all newborns with orofacial clefts should undergo genetic testing, at least with an appropriate gene panel for CLP; only after negative results, and where there is no suggestive family history or additional clinical symptoms (which validate the requirement for additional exome or genome-wide investigations), should a multifactorial risk prediction percentage be provided for families.

## Data Availability

The datasets presented in this study can be found in online repositories. The names of the repository/repositories and accession number(s) can be found below: https://www.lovd.nl, 0000816624, 0000816623, 0000816622. Accession: E-MTAB-11360. https://www.ebi.ac.uk/arrayexpress/experiments/E-MTAB-11360.
