# Supplementary material for: Monogenic Versus Multifactorial Inheritance in the Development of Isolated Cleft Palate: A Whole Genome Sequencing Study
Source: Front Genet. 2022 Feb 24;13:828534. doi: 10.3389/fgene.2022.828534 (PMC8907258; doi:10.3389/fgene.2022.828534)
Supplement: Supplementary file 1 [file DataSheet1.docx]

Supplement

Table 1. Selected list of genes

| Gene ID | Symbol | Gene ID | Symbol | Gene ID | Symbol | Gene ID | Symbol |
| --- | --- | --- | --- | --- | --- | --- | --- |
| "ENSG00000075624" | "ACTB" | "ENSG00000152223" | "EPG5" | "ENSG00000104973" | "MED25" | "ENSG00000108055" | "SMC3" |
| "ENSG00000184009" | "ACTG1" | "ENSG00000171320" | "ESCO2" | "ENSG00000134138" | "MEIS2" | "ENSG00000105771" | "SMG9" |
| "ENSG00000180318" | "ALX1" | "ENSG00000103067" | "ESRP2" | "ENSG00000005102" | "MEOX1" | "ENSG00000102172" | "SMS" |
| "ENSG00000156150" | "ALX3" | "ENSG00000104313" | "EYA1" | "ENSG00000101871" | "MID1" | "ENSG00000125835" | "SNRPB" |
| "ENSG00000184675" | "AMER1" | "ENSG00000177706" | "FAM20C" | "ENSG00000011143" | "MKS1" | "ENSG00000159140" | "SON" |
| "ENSG00000167522" | "ANKRD11" | "ENSG00000138081" | "FBXO11" | "ENSG00000163132" | "MSX1" | "ENSG00000125398" | "SOX9" |
| "ENSG00000137962" | "ARHGAP29" | "ENSG00000102302" | "FGD1" | "ENSG00000187616" | "MYMK" | "ENSG00000100014" | "SPECC1L" |
| "ENSG00000031081" | "ARHGAP31" | "ENSG00000107831" | "FGF8" | "ENSG00000110400" | "NECTIN1" | "ENSG00000124356" | "STAMBP" |
| "ENSG00000171456" | "ASXL1" | "ENSG00000077782" | "FGFR1" | "ENSG00000049759" | "NEDD4L" | "ENSG00000184058" | "TBX1" |
| "ENSG00000175054" | "ATR" | "ENSG00000066468" | "FGFR2" | "ENSG00000137601" | "NEK1" | "ENSG00000092607" | "TBX15" |
| "ENSG00000176022" | "B3GALT6" | "ENSG00000160867" | "FGFR4" | "ENSG00000164190" | "NIPBL" | "ENSG00000122145" | "TBX22" |
| "ENSG00000187676" | "B3GLCT" | "ENSG00000196924" | "FLNA" | "ENSG00000148400" | "NOTCH1" | "ENSG00000070814" | "TCOF1" |
| "ENSG00000027847" | "B4GALT7" | "ENSG00000136068" | "FLNB" | "ENSG00000046651" | "OFD1" | "ENSG00000119977" | "TCTN3" |
| "ENSG00000183337" | "BCOR" | "ENSG00000176692" | "FOXC2" | "ENSG00000135903" | "PAX3" | "ENSG00000100726" | "TELO2" |
| "ENSG00000125845" | "BMP2" | "ENSG00000128573" | "FOXP2" | "ENSG00000172943" | "PHF8" | "ENSG00000137203" | "TFAP2A" |
| "ENSG00000156970" | "BUB1B" | "ENSG00000138759" | "FRAS1" | "ENSG00000092621" | "PHGDH" | "ENSG00000088451" | "TGDS" |
| "ENSG00000168014" | "C2CD3" | "ENSG00000134363" | "FST" | "ENSG00000154864" | "PIEZO2" | "ENSG00000163235" | "TGFA" |
| "ENSG00000048342" | "CC2D2A" | "ENSG00000160282" | "FTCD" | "ENSG00000197563" | "PIGN" | "ENSG00000092969" | "TGFB2" |
| "ENSG00000093009" | "CDC45" | "ENSG00000140718" | "FTO" | "ENSG00000060642" | "PIGV" | "ENSG00000119699" | "TGFB3" |
| "ENSG00000039068" | "CDH1" | "ENSG00000107485" | "GATA3" | "ENSG00000101333" | "PLCB4" | "ENSG00000106799" | "TGFBR1" |
| "ENSG00000129757" | "CDKN1C" | "ENSG00000135414" | "GDF11" | "ENSG00000052126" | "PLEKHA5" | "ENSG00000163513" | "TGFBR2" |
| "ENSG00000171316" | "CHD7" | "ENSG00000152661" | "GJA1" | "ENSG00000166689" | "PLEKHA7" | "ENSG00000143183" | "TMCO1" |
| "ENSG00000196811" | "CHRNG" | "ENSG00000106571" | "GLI3" | "ENSG00000068654" | "POLR1A" | "ENSG00000073282" | "TP63" |
| "ENSG00000169105" | "CHST14" | "ENSG00000078369" | "GNB1" | "ENSG00000125630" | "POLR1B" | "ENSG00000167632" | "TRAPPC9" |
| "ENSG00000060718" | "COL11A1" | "ENSG00000147257" | "GPC3" | "ENSG00000171453" | "POLR1C" | "ENSG00000108395" | "TRIM37" |
| "ENSG00000204248" | "COL11A2" | "ENSG00000158055" | "GRHL3" | "ENSG00000186184" | "POLR1D" | "ENSG00000104447" | "TRPS1" |
| "ENSG00000139219" | "COL2A1" | "ENSG00000147099" | "HDAC8" | "ENSG00000102312" | "PORCN" | "ENSG00000123607" | "TTC21B" |
| "ENSG00000112280" | "COL9A1" | "ENSG00000068001" | "HYAL2" | "ENSG00000185920" | "PTCH1" | "ENSG00000196230" | "TUBB" |
| "ENSG00000049089" | "COL9A2" | "ENSG00000198331" | "HYLS1" | "ENSG00000077092" | "RARB" | "ENSG00000141759" | "TXNL4A" |
| "ENSG00000184374" | "COLEC10" | "ENSG00000112144" | "ICK" | "ENSG00000182872" | "RBM10" | "ENSG00000124486" | "USP9X" |
| "ENSG00000118004" | "COLEC11" | "ENSG00000187535" | "IFT140" | "ENSG00000168214" | "RBPJ" | "ENSG00000157796" | "WDR19" |
| "ENSG00000197603" | "CPLANE1" | "ENSG00000138002" | "IFT172" | "ENSG00000142599" | "RERE" | "ENSG00000119333" | "WDR34" |
| "ENSG00000102974" | "CTCF" | "ENSG00000101052" | "IFT52" | "ENSG00000169071" | "ROR2" | "ENSG00000118965" | "WDR35" |
| "ENSG00000198561" | "CTNND1" | "ENSG00000068885" | "IFT80" | "ENSG00000122406" | "RPL5" | "ENSG00000126870" | "WDR60" |
| "ENSG00000215301" | "DDX3X" | "ENSG00000104331" | "IMPAD1" | "ENSG00000197728" | "RPS26" | "ENSG00000114251" | "WNT5A" |
| "ENSG00000118197" | "DDX59" | "ENSG00000117595" | "IRF6" | "ENSG00000233927" | "RPS28" | "ENSG00000103489" | "XYLT1" |
| "ENSG00000172893" | "DHCR7" | "ENSG00000172977" | "KAT5" | "ENSG00000196218" | "RYR1" | "ENSG00000112365" | "ZBTB24" |
| "ENSG00000102967" | "DHODH" | "ENSG00000083168" | "KAT6A" | "ENSG00000101115" | "SALL4" | "ENSG00000169554" | "ZEB2" |
| "ENSG00000128917" | "DLL4" | "ENSG00000123700" | "KCNJ2" | "ENSG00000119042" | "SATB2" | "ENSG00000043355" | "ZIC2" |
| "ENSG00000108813" | "DLX4" | "ENSG00000004487" | "KDM1A" | "ENSG00000244486" | "SCARF2" | "ENSG00000156925" | "ZIC3" |
| "ENSG00000130158" | "DOCK6" | "ENSG00000147050" | "KDM6A" | "ENSG00000100934" | "SEC23A" | "ENSG00000084073" | "ZMPSTE24" |
| "ENSG00000107404" | "DVL1" | "ENSG00000100578" | "KIAA0586" | "ENSG00000184640" | "SEPT9" | "ENSG00000130449" | "ZSWIM6" |
| "ENSG00000161202" | "DVL3" | "ENSG00000198954" | "KIF1BP" | "ENSG00000143368" | "SF3B4" |  |  |
| "ENSG00000187240" | "DYNC2H1" | "ENSG00000166813" | "KIF7" | "ENSG00000164690" | "SHH" |  |  |
| "ENSG00000138036" | "DYNC2LI1" | "ENSG00000167548" | "KMT2D" | "ENSG00000126778" | "SIX1" |  |  |
| "ENSG00000147155" | "EBP" | "ENSG00000160789" | "LMNA" | "ENSG00000138083" | "SIX3" |  |  |
| "ENSG00000151617" | "EDNRA" | "ENSG00000135341" | "MAP3K7" | "ENSG00000177045" | "SIX5" |  |  |
| "ENSG00000090776" | "EFNB1" | "ENSG00000166974" | "MAPRE2" | "ENSG00000157933" | "SKI" |  |  |
| "ENSG00000108883" | "EFTUD2” | "ENSG00000127241" | "MASP1" | "ENSG00000155850" | "SLC26A2" |  |  |
| "ENSG00000130741" | "EIF2S3" | "ENSG00000012174" | "MBTPS2" | "ENSG00000166949" | "SMAD3" |  |  |
| "ENSG00000141543" | "EIF4A3" | "ENSG00000184634" | "MED12" | "ENSG00000141646" | "SMAD4" |  |  |
| "ENSG00000163378" | "EOGT" | "ENSG00000123066" | "MED13L" | "ENSG00000072501" | "SMC1A" |  |  |

Table 2. Minor allele frequencies (MAF) of cleft palate Latvian cohort compared to gnomAD European non-Finnish population. Single nucleotide polymorphism selection from Mukhopadhyay, 2020 study.

| Site | SNP ID | MAF  CP - LV data  Current study | MAF  gnomAD |
| --- | --- | --- | --- |
| 1p36.13 | rs78998514 | 0,43 | 0.364 |
| 2p25.3 | rs1362227148 | 0,00 | 0.0001033 |
| 2p24.3 | rs36094286 | 0,00 | SNP ID does not exist |
| 2q14.1 | chr2:113,497,779 | 0.136 | 0.056 |
| 2q35 | rs1164161401 | 0,00 | 0.09342 |
| 5q11.2 | rs1290483247 | 0,00 | low quality site |
| 6p22.2 | rs1747567 | 0,00 | low quality site |
| 6q25.3 | chr6:157,311,140 | 0.136 | 0.17 |
| 8q24.3 | rs72728755 | 0.136 | multiallelic variant |
| 8q24.3 | rs1429661747 | 0,00 | SNP ID does not exist |
| 9p11.2 | rs1471353675 | 0,00 | 0,00 |
| 9q34.2 | rs879409092 | 0,00 | SNP ID does not exist |
| 12p13.32 | rs1293776695 | 0,00 | SNP ID does not exist |
| 12p13.31 | rs1463969293 | 0,00 | 0.1176 |
| 17p11.2 | rs1446333119 | 0,00 | low quality site |
| 18p11.21 | rs576835177 | 0,00 | 0.0 |
| 18q23 | rs1381043271 | 0,00 | 0.1351 |
| 20q11.1 | rs1321001584 | 0,00 | SNP ID does not exist |
| 21q22.3 | rs2839575 | 0.613 | 0.2749 |
| Xq28 | rs306890 | 0,00 | 0.2509 |
